# The fine-tuning of endoplasmic reticulum stress response and autophagy activation during trophoblast syncytialization

**DOI:** 10.1038/s41419-019-1905-6

**Published:** 2019-09-09

**Authors:** Daniel Bastida-Ruiz, Lucile Yart, Christine Wuillemin, Pascale Ribaux, Nolwenn Morris, Manuella Epiney, Begoña Martinez de Tejada, Marie Cohen

**Affiliations:** Department of Pediatrics, Gynecology and Obstetrics, Faculty of Medicine, University of Geneva, 1206 Geneva, Switzerland

**Keywords:** Chaperone-mediated autophagy, Differentiation

## Abstract

The syncytiotrophoblast (STB) is a multinuclear layer forming the outer surface of the fetal part of the placenta deriving from villous cytotrophoblastic cell (vCTB) fusion and differentiation. This syncytialization process is characterized by morphological and biochemical alterations of the trophoblast, which probably require removal of pre-existing structures and proteins to maintain cell homeostasis and survival. Interestingly, autophagy, which allows degradation and recycling of cellular components, was shown to be activated in syncytiotrophoblast. Here we examined the involvement of endoplasmic reticulum stress (ERS) response in autophagy activation during vCTB syncytialization. We first demonstrated the activation of ERS response and autophagy during the time course of trophoblastic cell fusion and differentiation. Alteration of autophagy activation in vCTB by chemical treatments or Beclin-1 expression modulation leads to a decrease in trophoblastic syncytialization. Furthermore, ERS response inhibition by chemical treatment or siRNA strategy leads to a default in syncytialization, associated with alteration of autophagy markers and cell survival. From these data, we suggest that ERS response, by fine regulation of autophagy activation, may serve as an adaptive mechanism to promote cell survival during trophoblastic syncytialization.

## Introduction

The placenta is a highly specialized transient organ that protects, oxygenizes, and nourishes the developing fetus by linking maternal and fetal circulation^1^. Highly vascularized structures, referred to as chorionic villi, are involved in the efficient exchange of different molecules necessary for fetal development^2^. The formation of these complex multilayered structures is orchestrated by cytotrophoblastic cells (CTB)^3^. The mature chorionic villi are covered by an essential multinucleated continuous monolayer, the syncytiotrophoblast (STB), which is in direct contact with the maternal blood and functions as a nutrients and gas carrier, hormone secretion organ (β-human chorionic gonadotropin (β-hCG), steroids, etc.), and immunological barrier^2^. Underlying the STB reside mononucleated villous CTB (vCTB), which participate in the STB cellular turnover by asymmetric division. Indeed, asymmetric division generates one proliferative daughter cell that will continue dividing, and a second daughter cell, which will fuse and differentiate into the STB^4^. The multistep process where vCTB, through their ability to fuse and differentiate, give rise to the final multinucleated mature STB, is called syncytialization and it is crucial for placentation.

vCTB fusion has to be tightly regulated since a limited fusion rate may lead to an abnormal STB layer formation and placental function deficit, whereas an accelerated fusion process would lead to the same phenotype due to a too rapid depletion of the regenerative pool of vCTB^5^.

The mechanisms involved in syncytialization are still unclear; however, several factors implicated in this process have been identified. The expression of fusogenic proteins, such as syncytin 1, syncytin 2, and their receptors^5,6^, or the phosphatidylserine enrichment at the vCTB surface due to caspase 8 activation^7,8^ are known to be required for STB formation. Moreover, caspase 8 activation is also involved in the cytoskeleton rearrangement of CTB observed during syncytialization^7,8^. In addition, autophagy activation was observed in the STB^9,10^ and a positive correlation between syncytialization and autophagy has been reported in the choriocarcinoma cell line, BeWo^11^. It has been hypothesized that autophagy activation in vCTB would serve to provide energy to the cells in situations of moderate nutrient depletion and/or oxidative stress^10^. Furthermore, autophagy may represent a pivotal mechanism for organelle reorganization or degradation required after incorporation of cytoplasmic contents of the fusing of vCTB into the syncytium as observed during myotube formation^12^. Several conditions or factors such as nutrient signaling, oxidative stress, or endoplasmic reticulum stress (ERS) may be involved in vCTB autophagy activation, as it was observed in other cell types^13^. ERS response (or unfolded protein response (UPR)) is an adaptive mechanism that is activated in cells suffering from ERS in order to overcome this situation. In homeostatic conditions, the UPR pathways are maintained in an inactive state due to glucose-regulated protein 78 (GRP78) binding to the three UPR-associated proteins: inositol-requiring enzyme 1α (IRE1α), protein kinase RNA-like endoplasmic reticulum kinase (PERK), and activating transcription factor 6 (ATF6) [review^14^]. GRP78 is a molecular chaperone with higher affinity for unfolded proteins than for UPR-related proteins and, under ERS conditions, assists in the correct folding of proteins in the ER lumen, which implies its disconnection from the UPR-associated protein complexes. The dissociation of GRP78 from the UPR-related proteins confers to the remaining ones the ability to become active, thereby inducing the UPR^14^. Interestingly, GRP78 seems to play an important role in syncytialization, since reduced expression of GRP78 in trophoblastic cells by small interfering RNA (siRNA) leads to a decrease in cell fusion and differentiation^15^. However, the implications of the UPR in vCTB fusion and differentiation remain elusive. The classic consequences derived from the UPR activation include an increase in chaperones and ER-associated protein degradation-related protein expression and a decrease in general protein expression [review^14^]. In addition, the UPR could activate autophagy and apoptosis, two mechanisms that regulate cell fate and may play a critical role during placentation^16,17^. However, as mentioned before, the UPR and autophagy activation during syncytialization has not yet been documented. In this study, we have demonstrated that UPR and autophagy are directly implicated in vCTB cell fusion and differentiation. In addition, we have shown that modulation of UPR in vCTB purified from term placenta alters autophagy, cell fusion, differentiation, and cell survival, demonstrating that UPR activation is crucial for cell adaptation to the syncytialization process.

## Results

### UPR is activated during BeWo cell fusion and plays a role in syncytialization

**Fig. 1:**
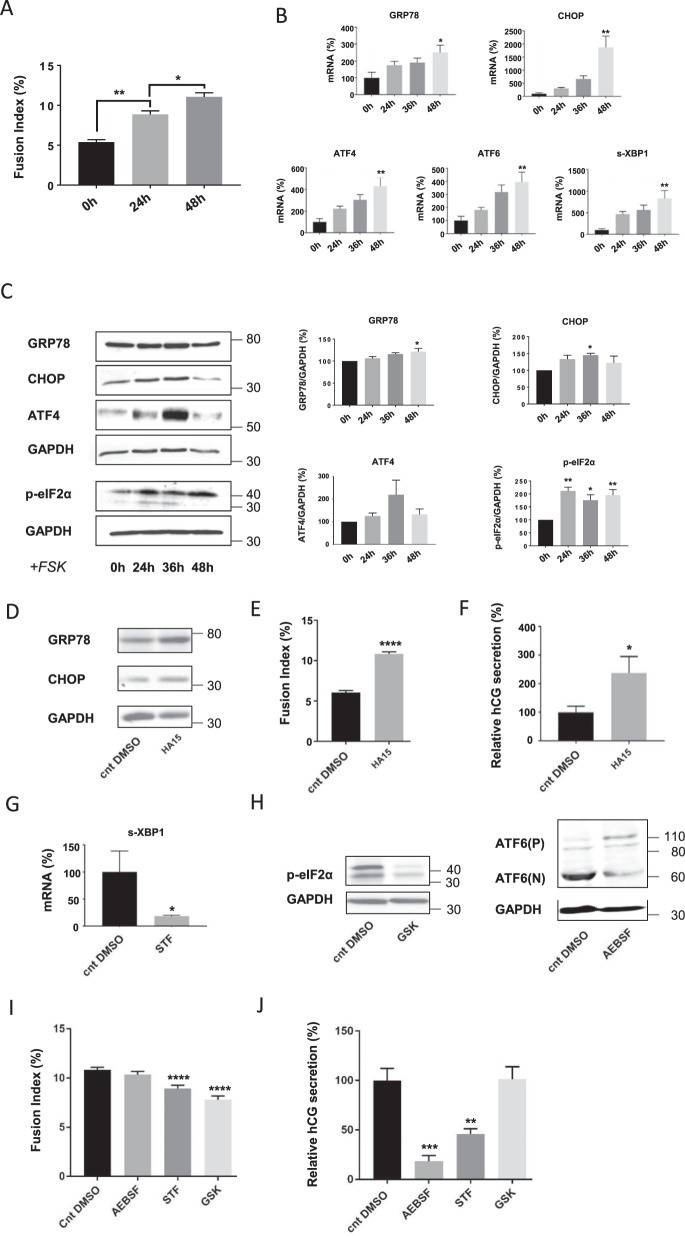
Role of unfolded protein response in cell fusion and differentiation of BeWo cells. **a**–**c** BeWo cells were seeded, and 24 h later treated with 100 µM Forskolin (FSK) for 24, 36 and 48 h. **a** Nuclei and syncytia were counted and fusion index was calculated. **b** RNA was retrotranscribed and 10 ng of cDNA were used to perform qPCR. **c**- Western blotting was performed on the cells. Proteins levels were quantified using the ImageJ software, and data are expressed as the fold change relative to the control. *n* = 3. Data represented as mean ± SEM. *P ≤ 0.05; **P ≤ 0.01; *t* test comparison test. **d**–**f** BeWo cells were treated 24 h after cell seeding with 10 µM HA15 for 48 h. **d** Western blotting was performed on the cells. **e** Nuclei and syncytia were counted and fusion index was calculated. **f** β-Human chorionic gonadotropin (β-hCG) was measured in culture supernatant by ELISA, normalized to the protein content and expressed relative to the control. *n* = 3. Data represented as mean ± SEM. *P ≤ 0.05; ****P ≤ 0.0001; *t* test comparison test. **g**–**j** BeWo cells were seeded, and 24 h later treated with 10 µM HA15 and 200 µM 4-(2-aminoethyl) benzenesulfonyl fluoride hydrochloride (AEBSF), 100 µM STF-083010 (STF), 100 nM GSK2656157 (GSK) or DMSO (Control DMSO, Cnt DMSO) for 48 h. **g** RNA was retrotranscribed and 10 ng of cDNA were used to perform qPCR. **h** Western blotting was performed on the cells. **i** Nuclei and syncytia were counted and fusion index was calculated. **j** β-Human chorionic gonadotropin (β-hCG) was measured in culture supernatant by ELISA, normalized to the protein content and expressed relative to the control. *n* = 3. Data represented as mean ± SEM. *P ≤ 0.05; ***P < 0.001; ****P ≤ 0.0001; *t* test comparison test

In order to investigate the implications of the UPR in syncytialization, we first used the human trophoblastic cell line model, BeWo, which is commonly used as a model to mimic syncytialization of placental villous trophoblast. In a forskolin (Fsk)-induced cell fusion time course, we initially examined the fusion index (FI), a measure that indicates the fusion rate of the cells. As expected under Fsk treatment, the FI was significantly increased over time (Fig. 1a). In parallel, the measurement of mRNA and/or protein expression of several UPR markers, including activating transcription factor 4 (ATF4), ATF6, the spliced form of X-box binding protein 1 (s-XBP1), GRP78, C/EBP homologous protein (CHOP), and the phosphorylated form of Eukaryotic translation initiation factor 2α (p-eIF2α), showed a general increased mRNA expression over time, which was significant at 48 h (Fig. 1b). The protein level of GRP78, CHOP, and p-eIF2α was significantly increased with the Fsk treatment, while ATF4 showed a non-significant tendency toward increased expression. These results suggest an activation of the UPR in BeWo cells during the cell fusion time course or Fsk treatment.

We then wanted to investigate whether the UPR activation can be a trigger of cell fusion and differentiation in BeWo cells. To achieve this objective, BeWo cells were treated in vitro with the chemical ERS inducer HA15 in an Fsk-free culture medium, and the FI was calculated after 48 h of treatment. The ER stress inducer increased the expression of the UPR-related proteins GRP78 and CHOP, confirming UPR activation (Fig. 1d). Interestingly, the FI was also augmented when the BeWo cells were treated with HA15 (Fig. 1e). In addition, measurement of the trophoblastic differentiation marker β-human chorionic gonadotropin (β-hCG) in a supernatant of BeWo cells culture demonstrated that the cell fusion increase reached by HA15 was accompanied by cell differentiation (Fig. 1f), suggesting that ERS can induce syncytialization. To demonstrate that the increased cell fusion and differentiation is due to UPR activation and not to side effects in the cells, we treated BeWo cells with HA15 and three UPR inhibitors: 4-(2-aminoethyl) benzenesulfonyl fluoride hydrochloride (AEBSF) that inhibits ATF6 activation, STF-083010 (STF) that prevents IRE1α activation, and GSK2656157 (GSK) that inhibits PERK. The inhibition of the different arms was controlled by measuring the protein or mRNA level of some specific branch-related proteins, such as s-XBP1 mRNA for STF (Fig. 1g), p-eIF2α for GSK, and ATF6 cleavage for AEBSF (Fig. 1h). A significant decrease in cell fusion was observed when BeWo cells were treated with IRE1α and PERK inhibitors (Fig. 1i). Moreover, a decrease in β-hCG secretion was detected when BeWo cells were treated with ATF6 and IRE1α pathways inhibitors (Fig. 1j). These results suggest that the UPR is not only activated but is also a trigger of BeWo syncytialization, reinforcing the importance of the UPR in placentation.

### UPR is activated during the vCTB cell fusion time course and is involved in syncytialization

**Fig. 2:**
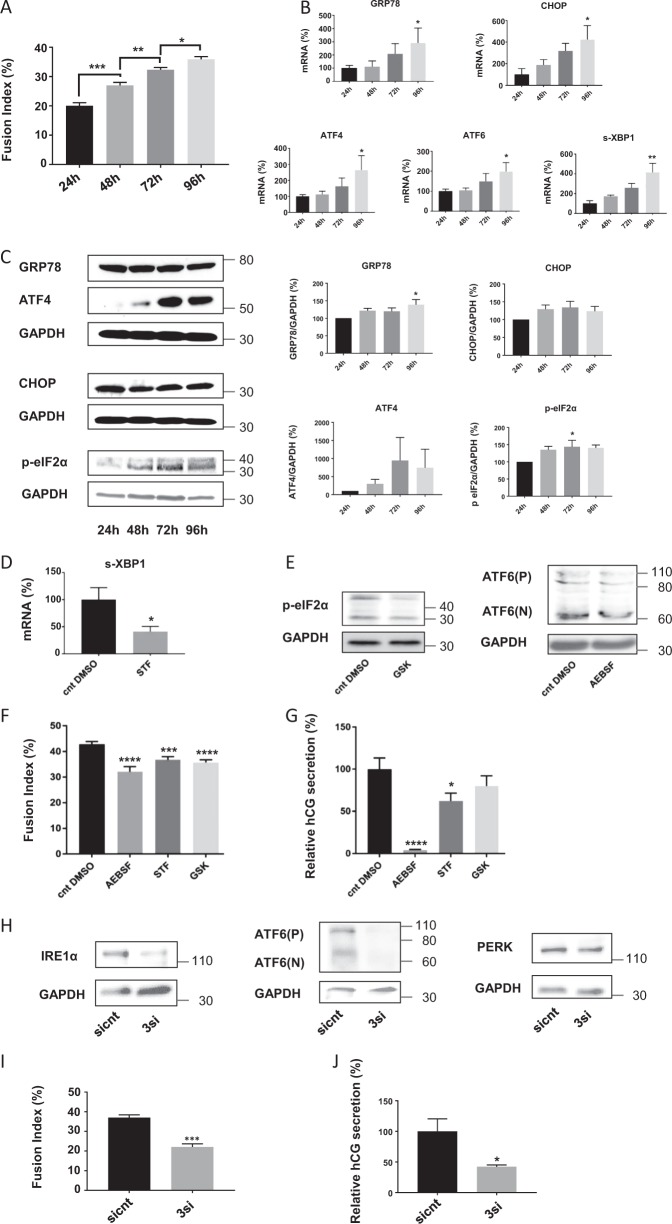
Role of unfolded protein response in cell fusion and differentiation of primary villous cytotrophoblastic cells. **a**–**c** Villous cytotrophoblastic (vCTB) cells were purified from human term placenta and seeded for 24, 48, 72, and 96 h. **a** Nuclei and syncytia were counted and fusion index was calculated. **b** RNA was retrotranscribed and 10 ng of cDNA were used to perform qPCR. *n* = 5. **c** Western blotting was performed on the cells. Proteins levels were quantified using the ImageJ software, and data are expressed as the fold change relative to 24 h of culture. *n* = 4. Data represented as mean ± SEM. *P ≤ 0.05; **P ≤ 0.01; *t* test comparison test. **d**–**g** vCTB were purified from human term placenta and seeded for 24 h prior treatment with 200 µM 4-(2-aminoethyl)benzenesulfonyl fluoride hydrochloride (AEBSF), 100 µM STF-083010 (STF), 100 nM GSK2656157 (GSK), or DMSO (Control DMSO, Cnt DMSO) for 48 h. **d** RNA was retrotranscribed and 10 ng of cDNA were used to perform qPCR. *n* = 4. **e** Western blotting was performed on the cells. *n* = 4. **f** Nuclei and syncytia were counted and fusion index was calculated. **g** β-Human chorionic gonadotropin (β-hCG) was measured in culture supernatant by ELISA, normalized to the protein content and expressed relative to the control. *n* = 3. Data represented as mean ± SEM. *P ≤ 0.05; ***P ≤ 0.001; ****P ≤ 0.0001; *t* test comparison test. **h**–**j** vCTB were purified from human placenta and seeded; 24 h later, cells were transfected with 16.6 nM of siATF6, 16.6 nM of siPERK, and 16.6 nM of siIRE1α (3si) or 50 nM of control siRNA (sicnt). **h** Western blotting was performed on the cells. *n* = 3. **i** Nuclei and syncytia were counted and fusion index was calculated. *n* = 3. **j** β-Human chorionic gonadotropin (β-hCG) was measured in culture supernatant by ELISA, normalized to the protein content and expressed relative to the control. *n* = 3. Data represented as mean ± SEM. *P ≤ 0.05; ***P ≤ 0.001 *t* test comparison test

The activation of the UPR was then investigated in human-purified term vCTB, which are able to spontaneously fuse in vitro. We first measured the FI of trophoblastic cells, observing a significant increase in cell fusion over time (Fig. 2a). The increased cell fusion was accompanied by a significant increase of the different UPR-related genes’ (ATF4, ATF6, s-XBP1, CHOP, GRP78) mRNA expression (Fig. 2b). A similar protein expression profile to the one observed in BeWo cells was found in the term trophoblastic cells; GRP78 and p-eIF2α expression was significantly increased at 96 and 72 h of culture, respectively, while CHOP and ATF4 showed a tendency toward the increase (Fig. 2c). The same tendency of UPR activation during trophoblast differentiation was also observed in first-trimester trophoblastic cells (Fig. S1).

To verify that ER stress is not just a result of the culture conditions, we evaluated ER stress markers in first-trimester trophoblast and term placenta in which STB is the predominant type of trophoblastic cells (Fig. S2). We thus confirmed that all the ER stress markers’ (except GRP78) expression levels are increased in term placenta compared to first-trimester trophoblast.

In order to confirm the implications of the UPR in the syncytialization process, we then treated purified vCTB cells with the different UPR inhibitors (AEBSF, STF, and GSK). Treatment efficacy was tested at the mRNA (Fig. 2d) or protein level of UPR markers (Fig. 2e). The inhibition of the three UPR pathways slightly, but significantly, decreased the vCTB fusion rate (Fig. 2f) and β-hCG secretion was significantly decreased when IRE1α and ATF6 pathways were inhibited (Fig. 2g). The different branches of the UPR are known to display an extensive cross-talk between them, compensating their modulation and showing interdependence^18,19^, as we can observe in trophoblastic cells (Fig. S3). This could explain the slight effects of the different UPR inhibitors on cell fusion. We thus decided to downregulate at the same time ATF6, IRE1α, and PERK expression in vCTB using siRNA strategy. The decrease in protein expression of these three UPR-related proteins was verified by western blot (Fig. 2h), and assessment of the FI (Fig. 2i) and β-hCG secretion (Fig. 2j) was performed on transfected cells, showing a significant decrease in both measures when the UPR was inhibited. These results suggest that the UPR is not only activated in vCTB but is also actively involved in the syncytialization process.

### Level of autophagy markers during the trophoblastic cell fusion time course

**Fig. 3:**
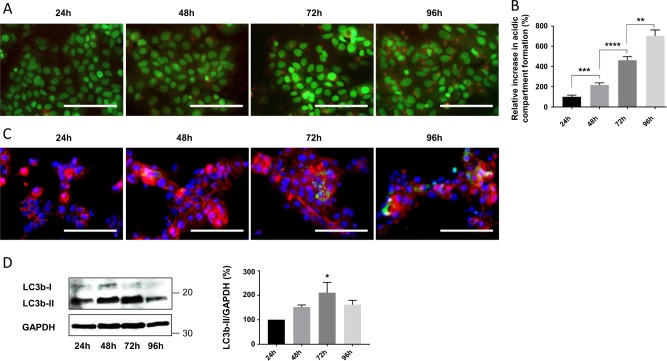
Activation of autophagy during syncytialization. **a** Primary trophoblastic cells were labeled with acridine orange 24, 48, 72 and 96 h after seeding and observed by fluorescence microscopy. Scale bar represents 100 μm. **b** Acridine orange quantification of the intensity and area of red signal, normalized to the total number of nuclei. *n* = 4. Data represented as mean ± SEM. **P ≤ 0.01; ***P ≤ 0.001; ****P ≤ 0.0001; *t* test comparison test. **c** Cells were labeled with anti-LC3b antibodies (green), anti-γ-catenin (red), and 4′,6-diamidino-2-phenylindole (DAPI) (nuclei, blue) and observed by fluorescence microscopy 24, 48, 72, and 96 h after seeding. Scale bar represents 100 μm. *n* = 3. **d** Western blotting was performed on the cells. LC3b-II and GAPDH levels were quantified using the ImageJ software, and data are expressed as the fold change relative to 24 h of culture. *n* = 4. Data represented as mean ± SEM. *P ≤ 0.05; ANOVA comparison test

The versatility of the UPR entails the activation of several processes, among them autophagy, which may promote cell survival during syncytialization. Interestingly, autophagy activity was observed during syncytialization^9^. In order to confirm the autophagy activation during vCTB cell fusion and differentiation, we first evaluated the expression of the autophagy marker microtubule-associated proteins 1A/1B light chain 3B (LC3b) and the presence of acidic compartment during a 96-h syncytialization time course. Live-cell staining was carried out at 24, 48, 72, and 96 h post-seeding with acridine orange, an organic dye that stains in red the acidic compartments and in green the cytoplasm and nucleus (Fig. 3a). Visual observation of the cells showed an increased red pixel intensity during the time course, which corresponds to an increased acidic compartment staining (Fig. 3a). Quantification and normalization of the results confirmed the increased acidic compartment formation during the time course of cell fusion (Fig. 3b). In parallel, immunolabeling of the autophagy marker LC3b showed an LC3b signal in trophoblastic cells, which was incremented during the time course of cell fusion (Fig. 3c). In addition, level of LC3bII was measured by western blot (Fig. 3d), displaying an increase in its expression during the time course of syncytialization, with a significant increase at 72 h (Fig. 3d). The increase in autophagy markers was also observed during first-trimester trophoblast differentiation (Fig. S4). Together, these results tend to confirm autophagy activation in trophoblastic cells during syncytialization.

### Autophagy is involved in trophoblastic syncytialization

**Fig. 4:**
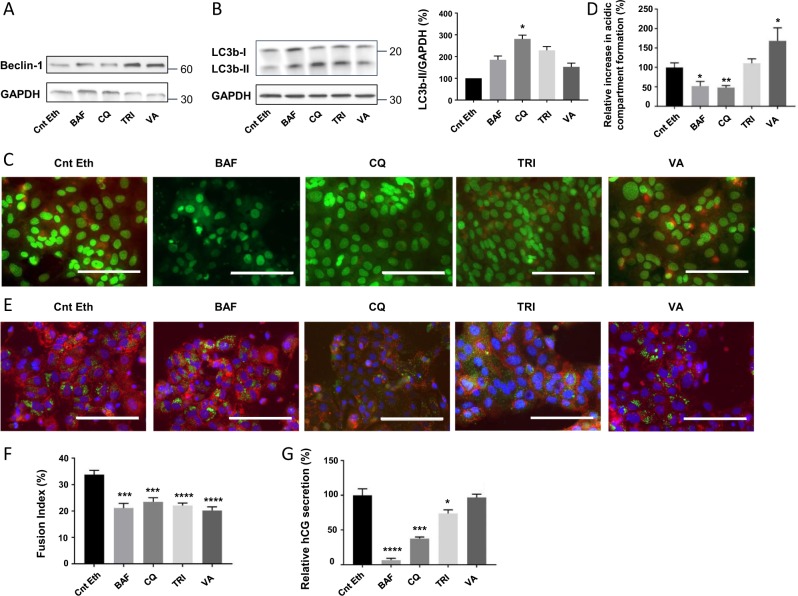
Consequences of autophagy modulation with autophagy inhibitors or activators on trophoblastic cell fusion and differentiation. vCTB were purified from human term placenta and seeded for 24 h prior treatment with 10 nM Bafilomycin A1 (BAF), 2 µM Chloroquine (CQ), 10 nM Trichostatin A (TRI), 200 µM Valproic acid (VA), or Ethanol (Control Ethanol, Cnt Eth) for 48 h. **a**, **b** Western blotting was performed on the cells. LC3b-II and GAPDH levels were quantified using the ImageJ software, and data are expressed as the fold change relative to the control. *n* = 4. Data represented as mean ± SEM. *P ≤ 0.05; ANOVA comparison test. **c** Cells were labeled with acridine orange 48 h post-treatment and observed by fluorescence microscopy. Scale bar represents 100 μm. **d** Acridine orange quantification of the intensity and area of red signal, normalized to the total number of nuclei. *n* = 4. Data represented as mean ± SEM. *P ≤ 0.05; **P ≤ 0.01; *t* test comparison test. **e** Trophoblastic cells were labeled with anti-LC3b antibodies (green), anti-γ-catenin (red), and 4′,6-diamidino-2-phenylindole (DAPI) (nuclei, blue) and observed by fluorescence microscopy 48 h post-treatment. Scale bar represents 100 μm. *n* = 3 **f** Nuclei and syncytia were counted and fusion index was calculated. *n* = 4. Data represented as mean ± SEM. ***P ≤ 0.001; ****P ≤ 0.0001; *t* test comparison test. **g** β-Human chorionic gonadotropin (β-hCG) was measured in culture supernatant by ELISA, normalized to the protein content and expressed relative to the control. *n* = 3. Data represented as mean ± SEM. *P ≤ 0.05; ***P ≤ 0.001; ****P ≤ 0.0001; *t* test comparison test

We hypothesized that autophagy could be activated during syncytialization to participate in the organelle reorganization/degradation required after the incorporation of the cytoplasmic content of the fusing of CTB into the syncytium. This process could be thus necessary to achieve cell fusion, differentiation and, finally, cell survival. To decipher the role of autophagy in the syncytialization process, we first evaluated the impact of autophagy modulation on syncytialization by treating human-purified vCTB with the autophagy inhibitors Bafilomycin A1 (BAF) and Chloroquine (CQ) or the autophagy activators Trichostatin A (TRI) and Valproic Acid (VA). Treatment efficacy was initially verified by quantifying the autophagy-initiator protein Beclin-1 expression level (Fig. 4a) and LC3bI and II expression levels (Fig. 4b). The inhibitors of autophagy maintained Beclin-1 protein expression, while the activators increased the expression of this autophagy initiator protein. As expected, we observed a significant increase in the activated form of LC3bII upon autophagy block confirming that autophagic flux is actually increased during syncytialization (Fig. 4b). BAF and CQ are known to act in the last stages of autophagy, more concretely blocking the acidification of already existing autophagosomes. The action mechanism of these drugs produces an accumulation of autophagosomes in the previous stages of acidification, explaining the increased LC3b signal in the cells upon autophagy inhibition treatment. Reasonably, upon acridine orange staining of vCTB cells, BAF and CQ treatment reduced the acidic compartment formation while VA significantly increased its formation (Fig. 4c, d). Furthermore, the LC3b signal detected by immunolabeling (Fig. 4e) showed an increased signal of LC3b with all the autophagy modulator treatments. Finally, all autophagy modulators altered the trophoblastic cell fusion (Fig. 4f) and all of them, except VA, altered hCG secretion (Fig. 4g).

**Fig. 5:**
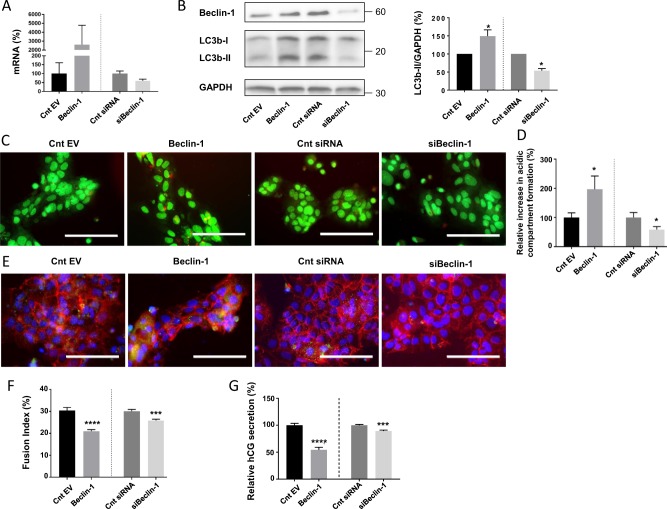
Consequences of autophagy modulation by Beclin-1 overexpression or silencing on syncytialization. **a**–**g** vCTB were purified from human term placenta and seeded for 24 h prior transfection with Cnt EV, Beclin-1, Cnt siRNA, or siBeclin-1 for 48 h. **a** RNA was retrotranscribed and 10 ng of cDNA were used to perform qPCR using primers for Beclin-1. *n* = 3. Data represented as mean ± SEM. **b** Western blotting was performed on the cells. LC3b-II and GAPDH levels were quantified using the ImageJ software, and data are expressed as the fold change relative to the control. *P ≤ 0.05; ***P ≤ 0.001; ANOVA comparison test. *n* = 3. **c** Cells were labeled with acridine orange 48 h post-transfection and observed by fluorescence microscopy. Scale bar represents 100 μm. **d** Acridine orange quantification of the intensity and area of red signal, normalized to the total number of nuclei. *n* = 3. Data represented as mean ± SEM. *P ≤ 0.05; *t* test comparison test. **e** Cells were labeled with anti-LC3b antibodies (green), anti-γ-catenin (red), and 4′,6-diamidino-2-phenylindole (DAPI) (nuclei, blue) and observed by fluorescence 48 h post-transfection. Scale bar represents 100 μm. **f** Nuclei and syncytia were counted and fusion index was calculated. *n* = 3. Data represented as mean ± SEM. ***P ≤ 0.001; ****P ≤ 0.0001; *t* test comparison test. **g** β-Human chorionic gonadotropin (β-hCG) was measured in culture supernatant by ELISA, normalized to the protein content and expressed relative to the control. *n* = 3. Data represented as mean ± SEM. ***P ≤ 0.001; ****P ≤ 0.0001; *t* test comparison test

In order to confirm the involvement of autophagy in syncytialization, we then modulated autophagy by changing Beclin-1 expression in vCTB. vCTB cells were transfected 24 h post-seeding either with a plasmid expressing the autophagy-initiator protein Beclin-1 or with siRNA-silencing Beclin-1. Beclin-1 mRNA (Fig. 5a) and proteins levels (Fig. 5b) were measured 48 h post-transfection, confirming increased Beclin-1 levels upon Beclin-1 plasmid transfection and decreased levels upon Beclin-1 silencing. To test the Beclin-1 transfection impact on autophagy activation, we evaluated the LC3b protein levels and the acidic compartment formation in vCTB cells. We observed an increase in the LC3bII level upon Beclin-1 transfection (Fig. 5b), and the opposite result upon Beclin-1 silencing (Fig. 5b), demonstrating that Beclin-1 modulation affects autophagy. Likewise, the immunostaining LC3b signal was either increased or decreased after transfection with Beclin-1 plasmid or after Beclin-1 silencing, respectively (Fig. 5e). In addition, acridine orange staining (Fig. 5c) and subsequent red pixel quantification (Fig. 5d) were performed 48 h post-transfection. The acridine orange normalized results showed an increase in acidic compartment formation in cells transfected with Beclin-1 plasmid and a decrease in acidic compartment formation in vCTB transfected with siBeclin-1 (Fig. 5c, d). In this way, autophagy induction or reduction caused by Beclin-1 plasmid or siBeclin-1 transfection was verified. Interestingly, the autophagy modulation led to a decrease in cell FI (Fig. 5f) and cell differentiation (Fig. 5g) under both conditions, supporting the involvement of autophagy in syncytialization and the importance of tight regulation of autophagy activation during this process.

### UPR is involved in increased level of autophagy markers during trophoblastic cell fusion and differentiation

**Fig. 6:**
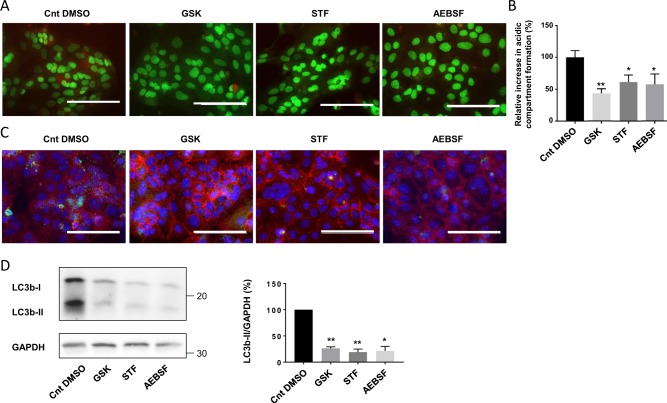
Effect of UPR modulation on autophagy activation in trophoblastic cells. vCTB were purified from human term placenta and seeded for 24 h prior treatment with 100 nM GSK2656157 (GSK), 100 µM STF-083010 (STF), 200 µM 4-(2-aminoethyl)benzenesulfonyl fluoride hydrochloride (AEBSF), or DMSO (Control DMSO, Cnt DMSO) for 48 h. **a** Cells were labeled with acridine orange 48 h post-treatment and observed by fluorescence microscopy. Scale bar represents 100 μm. **b** Acridine orange quantification of the intensity and area of red signal, normalized to the total number of nuclei. *n* = 4. Data represented as mean ± SEM. *P ≤ 0.05; **P ≤ 0.01; *t* test comparison test. **c** Cells were labeled with anti-LC3b antibodies (green), anti-γ-catenin (red), and 4′,6-diamidino-2-phenylindole (DAPI) (nuclei, blue) and observed by fluorescence microscopy 48 h post-treatment. Scale bar represents 100 μm. **d** Western blotting was performed on the cells. LC3b-II and GAPDH levels were quantified using the ImageJ software, and data are expressed as the fold change relative to the control. *n* = 4. Data represented as mean ± SEM. *P ≤ 0.05; ANOVA comparison test

Autophagy is known to be one of the outputs of UPR, and interestingly, we demonstrated that both of these processes are involved in syncytialization. We have thus hypothesized that the UPR is implicated in the autophagy activation during syncytialization. To test this hypothesis, we then decided to observe the autophagy markers in human-purified vCTB that were treated with inhibitors of the different arms of the UPR. Acridine orange staining of trophoblastic cells showed a decreased acidic compartment formation with AEBSF, STF, and GSK treatment (Fig. 6a, b). In addition, immunolabeling of LC3b (Fig. 6c) showed a decreased signal for all the treated conditions. LC3b protein level (Fig. 6d) measurement was performed and showed a decreased expression of LC3bII with all the treatments and a decreased ratio upon STF and GSK treatment. These results suggested that the UPR might be implicated in the autophagy activation during vCTB syncytialization process.

### UPR-activated autophagy plays a role in cell survival during syncytialization

**Fig. 7:**
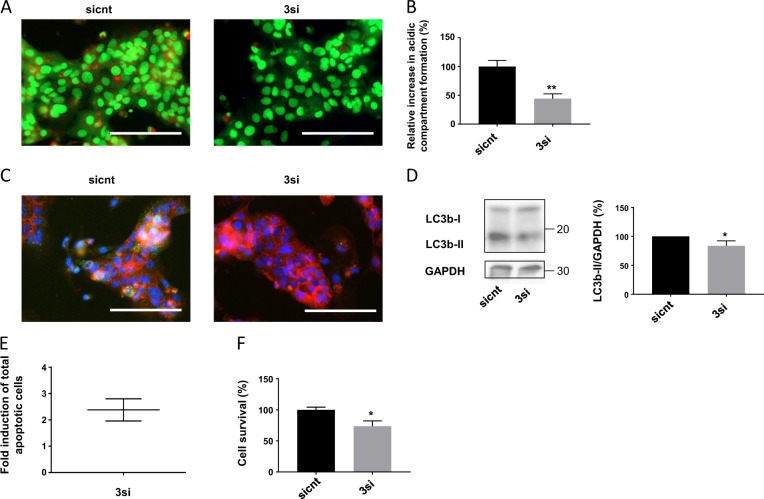
Role of UPR in autophagy activation and cell survival during syncytialization. **a**–**f** vCTB were purified from human term placenta. Twenty-four hours after seeding, vCTB cells were transfected with 16.6 nM of siATF6, 16.6 nM of siPERK, and 16.6 nM of siIRE1α (3si) or 50 nM of control siRNA (sicnt). **a** Cells were labeled with acridine orange 48 h post-transfection and observed by fluorescence microscopy. Scale bar represents 100 μm. **b** Acridine orange quantification of the intensity and area of red signal, normalized to the total number of nuclei. *n* = 3. Data represented as mean ± SEM. **P ≤ 0.01; *t* test comparison test. **c** Cells were labeled with anti-LC3b antibodies (green), anti-γ-catenin (red), and 4′,6-diamidino-2-phenylindole (DAPI) (nuclei, blue) and observed by fluorescence 48 h post-transfection. Scale bar represents 100 μm. **d** Western blotting was performed on the cells. LC3b-II and GAPDH levels were quantified using the ImageJ software, and data are expressed as the fold change relative to the control. *n* = 3. Data represented as mean ± SEM. *P ≤ 0.05; *t* test comparison test. **e** Flow cytometric analysis of trophoblastic cells and labeled with Annexin V-FITC and PI was performed 48 h after transfection. *n* = 3. **f** Cell viability was measured 120 h after transfection with the CellTiter-Glo Luminescent Cell Viability Assay. *n* = 3. Data represented as mean ± SEM. *P ≤ 0.05; *t* test comparison test

To confirm these results, we then decided to downregulate at the same time ATF6, IRE1α, and PERK in human-purified vCTB using the siRNA strategy, and autophagy markers were evaluated subsequently. First, acidic compartment formation was significantly decreased in transfected cells (Fig. 7a, b), together with LC3b immunolabeling (Fig. 7c) and LC3bII protein expression (Fig. 7d). Interestingly, UPR inhibition previously showed a decreased trophoblastic cell fusion (Fig. 2i) and differentiation (Fig. 2j) that correlates with decreased autophagy markers (Fig. 7a–d), supporting the link between the UPR, autophagy, and trophoblastic syncytialization.

Finally, we hypothesized that UPR-activated autophagy could be essential for a correct syncytialization due to its degradation capacity and its ability to promote cell survival. Annexin V and propidium iodide (PI) staining of cells, followed by a flow cytometric analysis, showed 2.4 times more apoptotic cells in triple siRNA-transfected vCTB cells than in the control cells (Fig. 7e). This result was consistent with the decreased cell survival that was observed upon UPR inactivation (Fig. 7f). We thus conclude that autophagy may be regulated by the UPR during the syncytialization process, therefore promoting cell survival.

## Discussion

Incorrect STB formation could lead to pregnancy disorders such as preeclampsia (PE), supporting the importance of syncytialization in placental development^20^. The UPR is able to induce apoptosis and autophagy^14^, which are required for trophoblastic syncytialization^9,16^. We thus hypothesized that UPR activation could be essential for correct cell fusion and differentiation.

In this study, we have demonstrated that the UPR is activated during cell fusion in both BeWo cells after induction of cell fusion with Fsk and in primary trophoblastic cells that display spontaneous cell fusion in vitro, suggesting a Fsk-independent UPR activation. We have also shown that preventing UPR activation by concomitant ATF6, PERK, and IRE1 downregulation causes a significant decrease in trophoblastic cell fusion and differentiation. However, the use of some chemical compounds to inhibit the different UPR pathways led to different effects on FI and hCG secretion. Indeed AEBSF treatment did not alter cell fusion but significantly decreased hCG secretion, suggesting that this molecule may modulate hCG expression or secretion independently of trophoblastic cell differentiation. In contrast, GSK treatment slightly altered cell fusion but not hCG secretion. This observation may be explained by a compensatory mechanism or by a direct effect of this drug on hCG expression. Moreover, the use of chemical UPR inhibitors only induced slight effects on cell fusion that may be due to the extensive cross-talk between the UPR branches, compensating their modulation and showing interdependence^18,19^.

It was previously reported that the UPR was essential for mammalian reproduction by allowing decidualization and placentation and that the lack of some UPR-related proteins caused default in embryo preimplantation or pregnancy disorders^21^. In addition, it was found that vascular endothelial growth factor A expression, which is necessary for a correct placenta vessel formation^22^, is regulated by the UPR-related proteins IRE1α, PERK, and ATF6^23^. Our results demonstrate that the UPR inhibition is also involved in cell fusion and differentiation, evidencing a new level of relevance of the UPR in placenta development.

Since the UPR has been reported to activate autophagy [review^14^], we hypothesized that the UPR can modulate autophagy during syncytialization, in order to degrade redundant organelles derived from cell fusion, providing additional energy to the cell under stress conditions, and promoting cell survival. We demonstrated that the inhibition of the UPR affects the autophagy activation, the syncytialization, and cell survival. Nevertheless, the role of autophagy in trophoblastic syncytialization remains unclear. It was recently shown that autophagy activation is enhanced during syncytialization^9^, as we have confirmed in this study. Interestingly, both autophagy induction and inhibition resulted in decreased cell fusion and differentiation. These results suggested that autophagy needed to be activated but more importantly, the autophagy activation should be tightly controlled to ensure syncytialization. An inhibition of autophagy could generate insufficient cell material recycling and energy supply, which may alter cell survival, as we observed upon UPR inhibition in vCTB. On the contrary, excessive activation of autophagy could lead to autosis, a non-apoptotic form of cell death, which is autophagy dependent and is triggered by an autophagy overactivation^24^.

Interestingly, the UPR^25^ and autophagy^26–30^ have been shown to be overactivated in early-onset PE placenta. We also confirmed the increased expression in both UPR and autophagy-related genes in PE vCTB (Fig. S5). In this way, the UPR stands out as a key point to further investigate in PE placenta in order to elucidate its actual role in the development of this condition. It was previously reported that disturbed vCTB fusion and STB formation were features of pregnancy disorders such as PE^31,32^. Therefore, in this article we bring forth new arguments supporting the contribution of overactivated UPR to the pathophysiology of early-onset PE, suggesting ERS inhibition as a possible target to improve these pregnancy pathology outcomes.

Finally, as was previously mentioned, vCTB are not the only cells capable of fusing to differentiate and become functional. More concretely, myoblasts, osteoclasts, and cancer cells are other human cell types able to perform these processes [review^33^]. The ability of these cells to fuse and differentiate leads us to speculate that similar mechanisms to those activated in vCTB could be implicated in these cell types. For instance, it was demonstrated that autophagy is increased and required during myoblast differentiation^12^, suggesting a possible translation of our model of cell–cell fusion to other cell types, and notably to tumor cells, which are frequently exposed to ERS.

## Materials and methods

### Ethics statement

The local ethics committee has approved this research. Before the inclusion in the study, informed written consent was obtained from all patients.

### Tissue collection

Collection of term and first-trimester placentae was done within 10 min after elective cesarean or elective pregnancy termination, respectively.

Five pieces of tissue were collected from different parts of the term placentae (*n* = 3) and treated separately in order to reduce variability.

First-trimester placentae were divided into two groups depending on the weeks of amenorrhea: early first trimester corresponding to 7–9 weeks of amenorrhea (*n* = 5) and late first trimester corresponding to 10–12 weeks of amenorrhea (*n* = 3). Tissues were immediately frozen at −80 °C after dissection.

### Tissue protein extraction

Tissues were immersed in 400 µl of protein lysis buffer and homogenized with a T10 basic ULTRA –TURRAX disperser (IKA Werke, Staufen im Breisgau, Germany). The supernatant containing the proteins was collected after centrifugation at 10,000 × *g*, 5 min, 4 °C.

### Tissue mRNA extraction

Tissues were grinded using a pestle and mortar previously cooled with liquid nitrogen. The grinded material was diluted in 300 µl of RNA lysis buffer and homogenized using QIAshredder homogenizer (QIAGEN, Hilden, Germany) as described by the manufacturer. Total RNA was extracted from the samples with the PureLinkRNA Mini Kit (AMBION, Austin, TX, USA).

### Purification of vCTB

vCTB were isolated from term normotensive and preeclamptic placentae and from first-trimester trophoblast. Purification took place according to the protocol described by Bischof et al.^34^. Briefly, small pieces of placental tissue were isolated and the tissue was enzymatically digested with a Difco Trypsin solution (BD, Le pont de Claix, France). After separation in a Percoll gradient (GE Healthcare, Uppsala, Sweden), the vCTB were immunopurified using monoclonal mouse anti-human CD45 immobilized antibodies (Dako, Glostrup, Denmark).

### Cell culture

BeWo cells (ATCC, CCL-98, Molsheim, France) were kindly provided by Dr. Thierry Fournier (INSERM U767, Paris, France) and cultured at 37 °C and 5% CO_2_ in Ham’s F12K medium (Gibco, Invitrogen, Basel, Switzerland) supplemented with 10% FBS (Biochrom AG, Oxoid AG, Basel, Switzerland) and 0.05 mg/ml gentamycin (Invitrogen, Basel, Switzerland). vCTB purified from placenta were cultured under the same conditions in Dulbecco’s modified Eagle’s medium (DMEM; Gibco, Invitrogen, Basel, Switzerland) supplemented with 10% FBS and 0.05 mg/ml gentamycin.

### Cell treatments

BeWo cells were treated 24 h post-seeding with 100 µM Forskolin (Sigma, St Louis, MO, USA) for 24, 36, and 48 h in order to induce syncytialization. ERS was also induced 24 h after cell seeding with 10 µM HA15 (Selleckchem, Zurich, Switzerland) for 48 h. To inhibit UPR, treatment was performed, 24 h post-seeding, by adding simultaneously the ERS inducer and the UPR inhibitors in the following concentrations: 10 µM HA15 and 200 µM AEBSF (Sigma, Darmstadt, Germany), 100 µM STF (Selleckchem, Zurich, Switzerland), or 100 nM GSK (Selleckchem, Zurich, Switzerland).

Purified vCTB were treated with 200 µM AEBSF, 100 µM STF, or 100 nM GSK 24 h after seeding to inhibit the UPR activation. The modulation of autophagy in vCTB was performed by adding 10 nM BAF (Invivogen, Toulouse, France), 2 µM CQ (Enzo Lifescience, Lausen, Switzerland), 10 nM TRI (Invivogen, Toulouse, France), or 200 µM VA (Invivogen, Toulouse, France) to the cell medium 24 h after seeding.

The different controls were performed by adding 24 h post-seeding the correspondent volume of DMSO (Sigma, St Louis, MO, USA) or ethanol (Sigma, St Louis, MO, USA) to the cells, depending on the vehicle of the treatment (DMSO for HA15, AEBSF, STF, and GSK; ethanol for BAF, CQ, TRI, and VA).

### Cell transfection

Two hundred and fifty thousand vCTB cells were seeded 24 h before transfection. JetPei transfection reagent was used to transfect cells following the manufacturer’s instructions (Polyplus transfection, Illkirch, France) using a plasmid expressing Beclin-1 (SantaCruz Biotechnology, Labforce, Switzerland) or an empty plasmid as a control (SantaCruz Biotechnology, Labforce, Switzerland). For experiments performed in a 6-well tissue culture plate (Falcon, Durham, NC, USA), 3 µg of the vectors were used; while 1 µg of the different vectors were used for experiments performed in µ-slide 8-well IbiTreat chamber slides (Ibidi GmbH, Martinsried, Gemany). For the Beclin-1-silencing experiments, transfection of 20 nM of siBeclin-1 (SantaCruz Biotechnology, Labforce, Switzerland) or control siRNA (SantaCruz Biotechnology, Labforce, Switzerland) was carried out using Interferin transfection reagent (Polyplus transfection Illkrich, France) and following the manufacturer’s protocol.

For the UPR silencing experiments, transfection of 16.6 nM of siATF6 (SantaCruz Biotechnology, Labforce, Switzerland), 16.6 nM of siIRE1α (SantaCruz Biotechnology, Labforce, Switzerland), and 16.6 nM of siPERK (SantaCruz Biotechnology, Labforce, Switzerland) or 50 nM control siRNA (SantaCruz Biotechnology, Labforce, Switzerland), was carried out using Interferin transfection reagent (Polyplus transfection Illkrich, France) and following the manufacturer’s protocol. Protein and mRNA extraction, immunofluorescence, or acridine orange staining were performed 48 h after cell transfection.

### Acridine orange staining

vCTB were seeded in µ-slide 8-well IbiTreat chamber slides (Ibidi GmbH, Martinsried, Germany) and cultured for 24, 48, 72, or 96 h. Cell culture medium was discarded and substituted by DMEM supplemented with 10% FBS, 0.05 mg/ml gentamycin, and 1 µg/ml acridine orange (Thermo Fisher Scientific, Eugene, Oregon USA). Cells were incubated at 37 °C for 30 min and the medium containing acridine orange was discarded and substituted by non-containing acridine orange growth medium. Three different images were acquired from different parts of each well by using an EVOS FL Cell Imaging System (ThermoFisher Scientific, Bothell, WA, USA). Picture analysis was performed using the ImageJ software. For each picture, acidic compartments (red staining) were assessed by quantification of the total red signal area (RA) and the mean intensity for red signal (MRI). A unique threshold was used to analyze all the pictures of one experiment. The total number of nuclei (*T*) was manually quantified (green staining). Data were calculated by using the following formula [(RA × MRI)/*T*].

### Western blot

Whole BeWo or vCTB cell extracts (40 µg of proteins) were fractionated by sodium dodecyl sulfate-polyacrylamide gel electrophoresis and transferred to nitrocellulose membrane for immunoblot analysis using rabbit anti-GRP78 antibodies (GL-19, 1:3,000 dilution from Sigma, Darmstadt, Germany), mouse anti-CHOP antibodies (sc-7351, 1:500 dilution from Santa Cruz Biotechnology, Heidelberg, Germany), rabbit anti-ATF4 antibodies (sc-200, 1:500 dilution from Santa Cruz Biotechnology, Heidelberg, Germany), rabbit anti-LC3b antibodies (3868S, 1:1,000 dilution from Cell Signaling), rabbit anti-p-eIF2α antibodies (9721 S, 1:1,000 dilution from Cell Signaling, Bioconcept, Allschwil, Switzerland), mouse anti-ATF6 antibodies (73–500, 1:1,000 dilution from BioAcademia, Osaka, Japan), rabbit anti-IRE1α (3294S, 1:1,000 dilution from Cell Signaling, Bioconcept, Allschwil, Switzerland), rabbit anti-PERK (3192S, 1:1,000 dilution from Cell Signaling, Bioconcept, Allschwil, Switzerland), rabbit anti-Beclin-1 antibodies (3495S, 1:1000 dilution from Cell Signaling, Bioconcept, Allschwil, Switzerland), and mouse anti-glyceraldehyde 3-phosphate dehydrogenase (anti-GAPDH) antibodies (MAB374, 1:10,000 dilution from Millipore, Temecula, CA, USA) as primary antibodies; also goat anti-mouse IgG-horseradish peroxidase (HRP) (sc-2005, 1:3,000 dilution from Santa Cruz Biotechnology, Heidelberg, Germany), and goat anti-rabbit IgG (H+L)-HRP conjugate (170–6515, 1:3,000 dilution from Bio-Rad, Basel, Switzerland) as secondary antibodies. Specific signal was detected using Amershan ECL Prime Western Blotting Detection Reagent (GE Healthcare, Buckinghamshire, UK) or Immobilon Western Chemiluminescent HPR Substrate (Billerica, MA, USA) and revealed with a PXi 6 chemiluminescence system (SynGene, UK). Bands were quantified using the ImageJ software.

### Immunofluorescence

vCTB were seeded in µ-slide 8-well IbiTreat chamber slides (Ibidi GmbH, Martinsried, Germany) and treated for 48 h as described in the “Cell treatments” section. Cells were then washed with phosphate-buffered saline (PBS) three times, fixed with 4% paraformaldehyde (Aldrich, Steinheim, Germany) for 10 min, and rewashed with PBS three times. Fixed cells were permeabilized with 0.2% Triton X100 (BioChemica Applichem, Darmstadt, Germany), diluted in PBS for 30 min at room temperature, and washed three times with PBS. Non-specific binding was blocked with 3% bovine serum albumin (BSA; Albumin Fraction V, PanReac Applichem, Barcelona, Spain) diluted in PBS for 30 min at room temperature. Cells were incubated with both rabbit anti-LC3b antibodies (3868 S, 1:200 dilution from Cell Signaling, Bioconcept, Allschwil, Switzerland) and mouse anti-γ-Catenin antibodies (13–8500, 1:200 dilution from Invitrogen, Basel, Switzerland), diluted in PBS–3% BSA overnight at 4 °C. Cells were then washed with PBS three times and incubated with secondary antibodies goat IgG–Chromeo 488 anti-rabbit (ab60314, dilution 1:1,000 from AbCam, Cambridge, UK) and goat IgG–Chromeo 642 anti-mouse (ab60318, dilution 1:500 from AbCam, Cambridge, UK), diluted in PBS–3% BSA for 2 h at room temperature. After washing three times with PBS in the dark, cells were incubated with 300 nM DAPI solution (Panreac AppliChem, Barcelona, Spain) for 10 minutes at room temperature in the dark. Finally, cells were washed three times with PBS in the dark and images were acquired with an EVOS FL Cell Imaging System (ThermoFisher scientific, Bothell, WA, USA).

### β-hCG measurement

Cell culture medium from BeWo cells and vCTB was collected and centrifuged for 5 min at 14,000 × *g* and the level of β-hCG was measured in the supernatant by enzyme-linked immunosorbent assay (DRG International, Diagnostik Medizintechnik, Oberdof, Switzerland) according to the manufacturer’s instructions. Results were normalized by total cellular protein content of corresponding wells and expressed relative to the control.

### Fusion index

Trophoblastic cell FI was determined by immunocytochemistry, as previously described^35^. Briefly, to visualize syncytia, cells were fixed in 3% paraformaldehyde and immunostained using mouse anti-desmoplakin antibodies (sc-390975, Santa Cruz Biotechnology, Heidelberg, Germany). Staining was revealed with diaminobenzidine (Dako, Carpinteria, CA, USA) after incubation with secondary antibodies anti-mouse IgG-HRP (sc-2005, Santa Cruz Biotechnology, Heidelberg, Germany). Images were acquired with an EVOS XL Core Cell Imaging System (ThermoFisher scientific, Bothell, WA, USA). FI expressed in percentage was calculated as follows: [(*N* − *S*)/*T*], where *N* equals the number of nuclei in syncytia, *S* equals the number of syncytia, and *T* equals the total number of nuclei counted. For each experiment, three different fields per well were analyzed. FI was calculated for at least three independent experiments, run in triplicate.

### Quantitative polymerase chain reaction (qPCR)

Total RNA was extracted from BeWo cells or vCTB using the PureLinkRNA Mini Kit (AMBION, Austin, TX, USA). Reverse transcription was performed with 1 µg of total RNA using the High Capacity cDNA Reverse Transcription Kit (Applied Biosystems, Life Technologies). The detection of the real-time qPCR product was performed using the KAPA SYBR FAST qPCR Kit Master Mix (Kapa Biosystems, Axon Lab, Baden, Switzerland) on an Eco Real-Time PCR System (Labgene Scientific, Châtel-St-Denis, Switzerland). The relative expression of the different genes was normalized to the three housekeeping genes GAPDH, Cyclophilin A, and HPRT1.

The oligonucleotide primers for qPCR were as described in Table 1.

**Table 1:** qPCR primers sequences

Gene name	Forward sequence	Reverse sequence
Syncytin1	5′-CCCAGGCGTTAGGTATACGA-3′	5′-GACCTTCCCTGAGGACTGTG-3′
Syncytin2	5′-CCTTCACTAGCAGCCTACCG-3′	5′-GCTGTCCCTGGTGTTTCAGT-3′
GRP78	5′-CGTGGAGATCATCGCCAAC-3′	5′-ACATAGGACGGCGTGATGC-3′
CHOP	5′-GCCTTTCTCCTTTGGGACACTGTCCAGC-3′	5′-CTCGGCGAGTCGCCTCTACTTCCC-3′
ATF4	5′-GTTCTCCAGCGACAAGGCTA-3′	5′-ATCCTGCTTGCTGTTGTTGG-3′
ATF6	5′-GAGTATTTTGTCCGCCTGCC-3′	5′-CGGGCTAAAAGGTGACTCCA-3′
Spliced XBP1	5′-CTGAGTCCGAATCAGGTGCAG-3′	5′-ATCCATGGGGAGATGTTCTGG-3′
LC3b	5′-ACACAGCATGGTCAGCGTCT-3′	5′-TTTCATCCCGAACGTCTCCT-3′
Beclin-1	5′-AACCTCAGCCGAAGACTGAA-3′	5′-GACGTTGAGCTGAGTGTCCA-3′
Cyclophilin A	5′-TACGGGTCCTGGCATCTTGT-3′	5′-CCATTTGTGTTGGGTCCAGC-3′
GAPDH	5′-CGACCACTTTGTCAAGCTCA-3′	5′-CCCTGTTGCTGTAGCCAAAT-3′
HPRT1	5′-ATGACCAGTCAACAGGGGAC-3′	5′-TGCCTGACCAAGGAAAGCAA-3′

### Annexin V/PI staining and flow cytometric analysis

vCTB cells were seeded in six-well plates and transfected as described in the “Cell transfection” section. Cells and medium were collected 120 h post-transfection and 200,000 cells were treated following the manufacturer’s protocol with the Annexin V-FITC Apoptosis Detection Kit (Sigma-Aldrich, St. Louis, MO, USA) prior to flow cytometric analysis.

### Cell survival measurement

vCTB cells were seeded in 96-well plates and transfected as described in the “Cell transfection” section. One hundred and twenty hours post-transfection, 50 µl of CellTiter-Glo Luminescent Cell Viability Assay (Promega, Madison, WI, USA) was added to each well. After 30 min of incubation in the dark, the well contents were transferred into white flat bottom 96-well plates (Sigma-Aldrich, Kennebunk, ME, USA) and luminescence was measured in GloMax 96-well Luminometer (EG501, Promega Biosystems Sunnyvale, Inc., Sunnyvale, CA, USA) with the Glomax 1.9.3 software.

### Statistics

Data were represented as means ± standard error of the mean (SEM) for at least three different samples. Statistical differences between samples were assessed by the Student’s *t* test or analysis of variance test as specified in each experiment and the *P* value <0.05 was considered significant. GraphPrism software was used to perform the different statistical analyses.

## Supplementary information

Supplemental Figure legends

Supplemental Figure 1

Supplemental Figure 2

Supplemental Figure 3

Supplemental Figure 4

Supplemental Figure 5
