# Supplementary material for: The fine-tuning of endoplasmic reticulum stress response and autophagy activation during trophoblast syncytialization
Source: Cell Death Dis. 2019 Sep 9;10(9):651. doi: 10.1038/s41419-019-1905-6 (PMC6733854; doi:10.1038/s41419-019-1905-6)
Supplement: Supplementary file 1 — Supplemental Figure legends [file 41419_2019_1905_MOESM1_ESM.docx]

**Figure S1. Unfolded protein response arms crosstalk**

vCTB cells were purified from human term placenta and seeded for 24hours prior treatment with 100 nM GSK2656157 (GSK), 100 µM STF-083010 (STF), 200 µM 4-(2-aminoethyl)benzenesulfonyl fluoride hydrochloride (AEBSF), or DMSO (Control DMSO, Cnt DMSO) for 48 hours. Western blotting was performed on the cells.

**Figure S2. Role of Unfolded Protein Response in cell fusion and differentiation of first trimester primary villous cytotrophoblastic cells.**

A-D Villous cytotrophoblastic (vCTB) cells were purified from first trimester trophoblast and seeded for 24, 48, 72 and 96 hours. A- Nuclei and syncytia were counted and fusion index was calculated n=4. B- β-human Chorionic Gonadotrophin (β-hCG) was measured in culture supernatant by ELISA, normalized to the protein content and expressed relative to the control. n=4. C- RNA was retrotranscribed and 10 ng of cDNA were used to perform qPCR. n=3. D- Western blotting was performed on the cells. Proteins levels were quantified using Image J software, and data are expressed as the fold change relative to 24h of culture. n=4. Data represented as mean ±SEM. *P≤0.05 ****P≤0.0001 t-test comparison test.

**Figure S3. UPR markers in first trimester and term placental tissues.**

A-B- RNA and proteins were extracted from human term placenta (n=3) and first trimester trophoblast (n=8) tissues. A- RNA was retrotranscribed and 10 ng of cDNA were used to perform qPCR. B- Western blotting was performed on tissues. Data represented as mean ±SEM. *P≤0.05 **P≤0.01 ***P≤0.001 ****P≤0.0001 t-test comparison test.

**Figure S4. Activation of autophagy during first trimester syncytialisation.**

A- First trimester primary trophoblastic cells were labeled with acridine orange 24, 48, 72 and 96 hours after seeding and observed by fluorescence microscopy. Scale bar represents 100 μm. B- Acridine orange quantification of the intensity and area of red signal, normalized to the total number of nuclei. n=4. Data represented as mean ±SEM. *P≤0.05 ***P≤0.001 ****P≤0.0001 t-test comparison test. C- Cells were labeled with anti-LC3b antibodies (green), anti-γ-catenin (red) and 4′,6-diamidino-2-phenylindole (DAPI) (nuclei, blue) and observed by fluorescence microscopy 24, 48, 72 and 96 hours after seeding. Scale bar represents 100 μm. n=4. D- Western blotting was performed on the cells. LC3b-II and GAPDH levels were quantified using Image J software, and data are expressed as the fold change relative to 24h of culture. n=3. Data represented as mean ±SEM.

**Figure S5. Unfolded protein response and autophagy mRNA expression in vCTB from normal pregnancy placenta and preeclamptic pregnancy placenta**

vCTB were purified from healthy pregnancy patients’ human placenta (cnt) or from preeclamptic pregnancy patients’ human placenta (PE) and seeded for 24 hours. RNA was retrotranscribed and 10 ng of cDNA were used to perform qPCR. n=6/group. Data represented as mean ±SEM. *P≤0.05 **P≤0.01 t-test comparison test.
