# Supplementary figures and images for: The fine-tuning of endoplasmic reticulum stress response and autophagy activation during trophoblast syncytialization
Source: Cell Death Dis. 2019 Sep 9;10(9):651. doi: 10.1038/s41419-019-1905-6 (PMC6733854; doi:10.1038/s41419-019-1905-6)

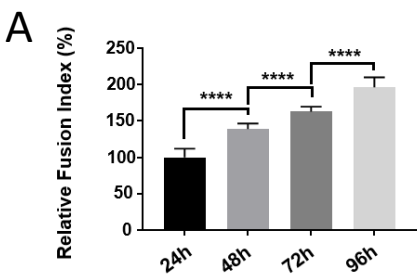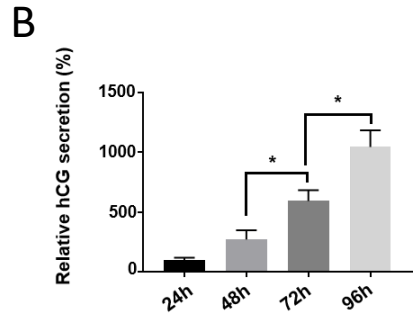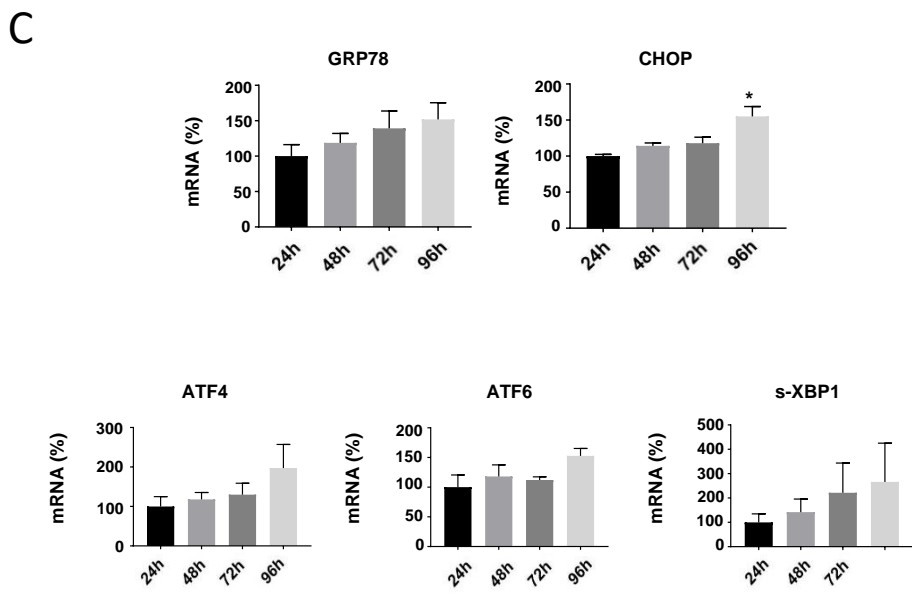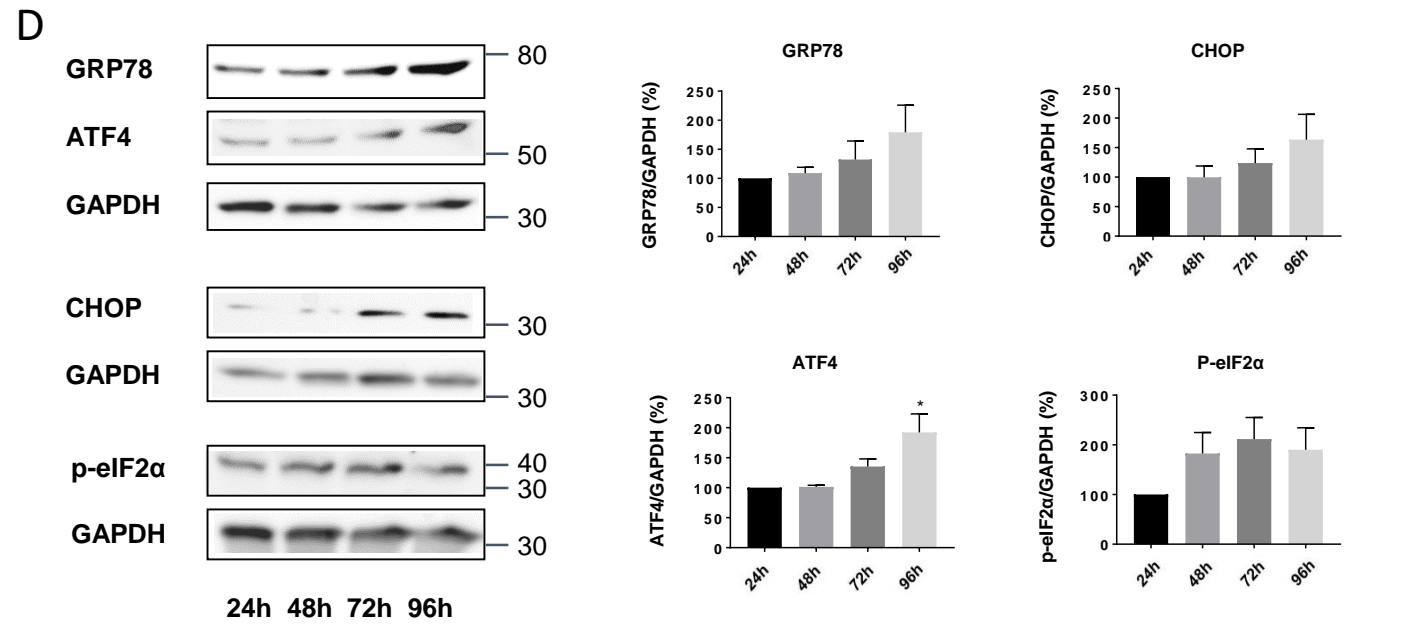

Figure S1

Supplement: Supplementary file 2 — Supplemental Figure 1 [file 41419_2019_1905_MOESM2_ESM.pdf]

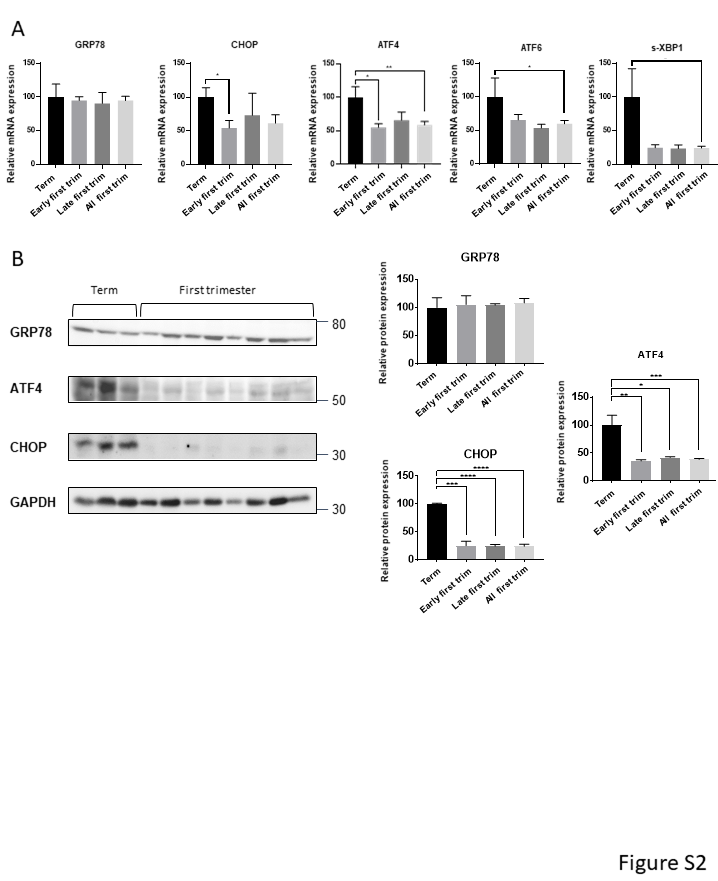

Supplement: Supplementary file 3 — Supplemental Figure 2 [file 41419_2019_1905_MOESM3_ESM.tif]

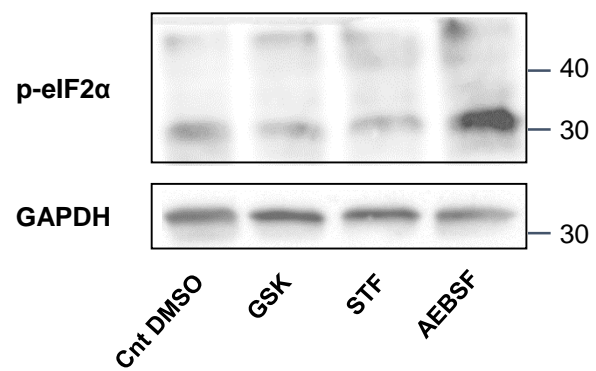

Figure S3

Supplement: Supplementary file 4 — Supplemental Figure 3 [file 41419_2019_1905_MOESM4_ESM.pdf]

A

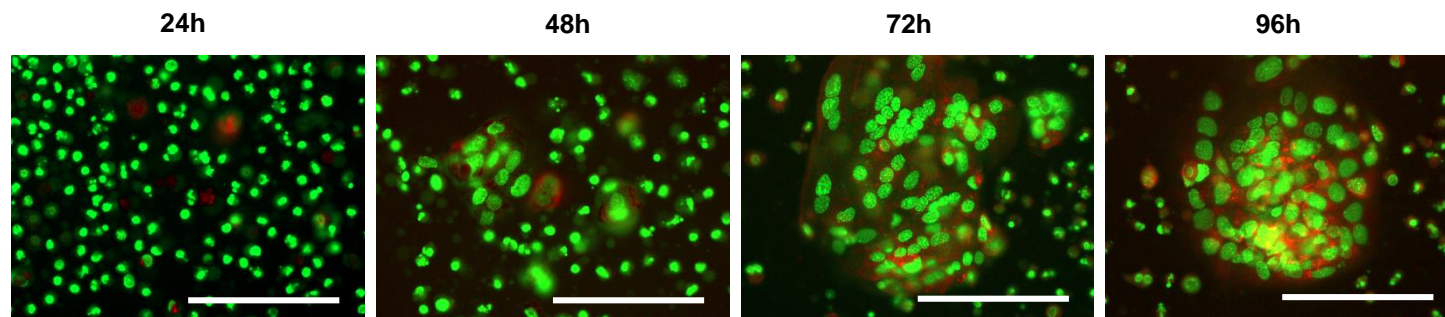

B

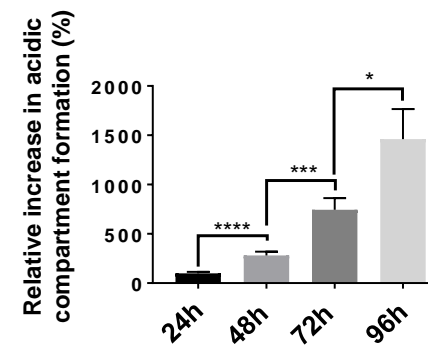

C

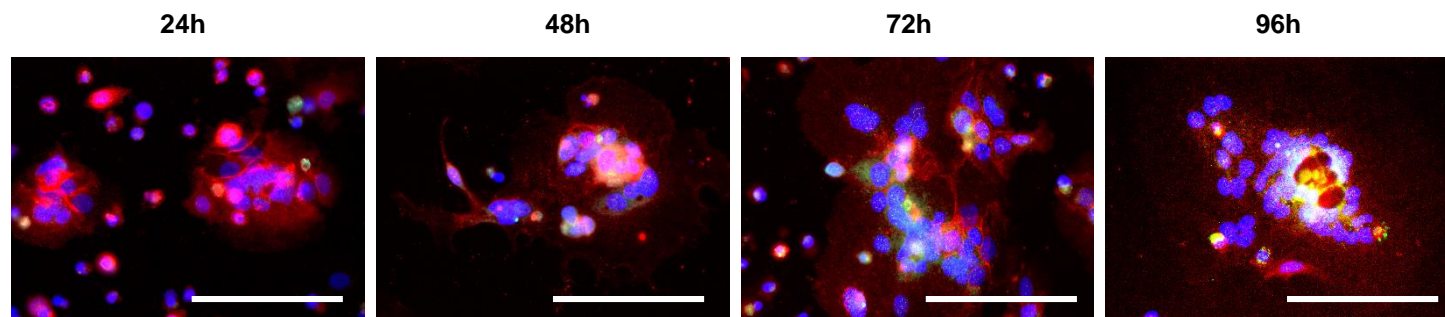

D

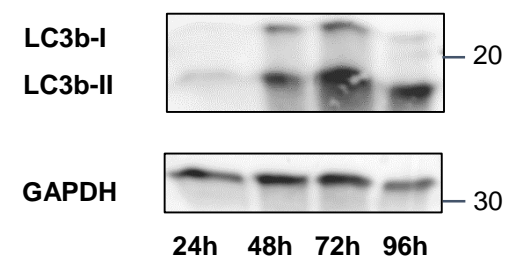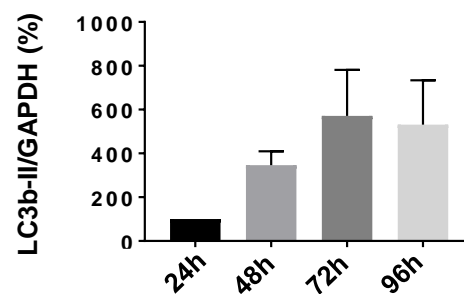

Figure S4

Supplement: Supplementary file 5 — Supplemental Figure 4 [file 41419_2019_1905_MOESM5_ESM.pdf]

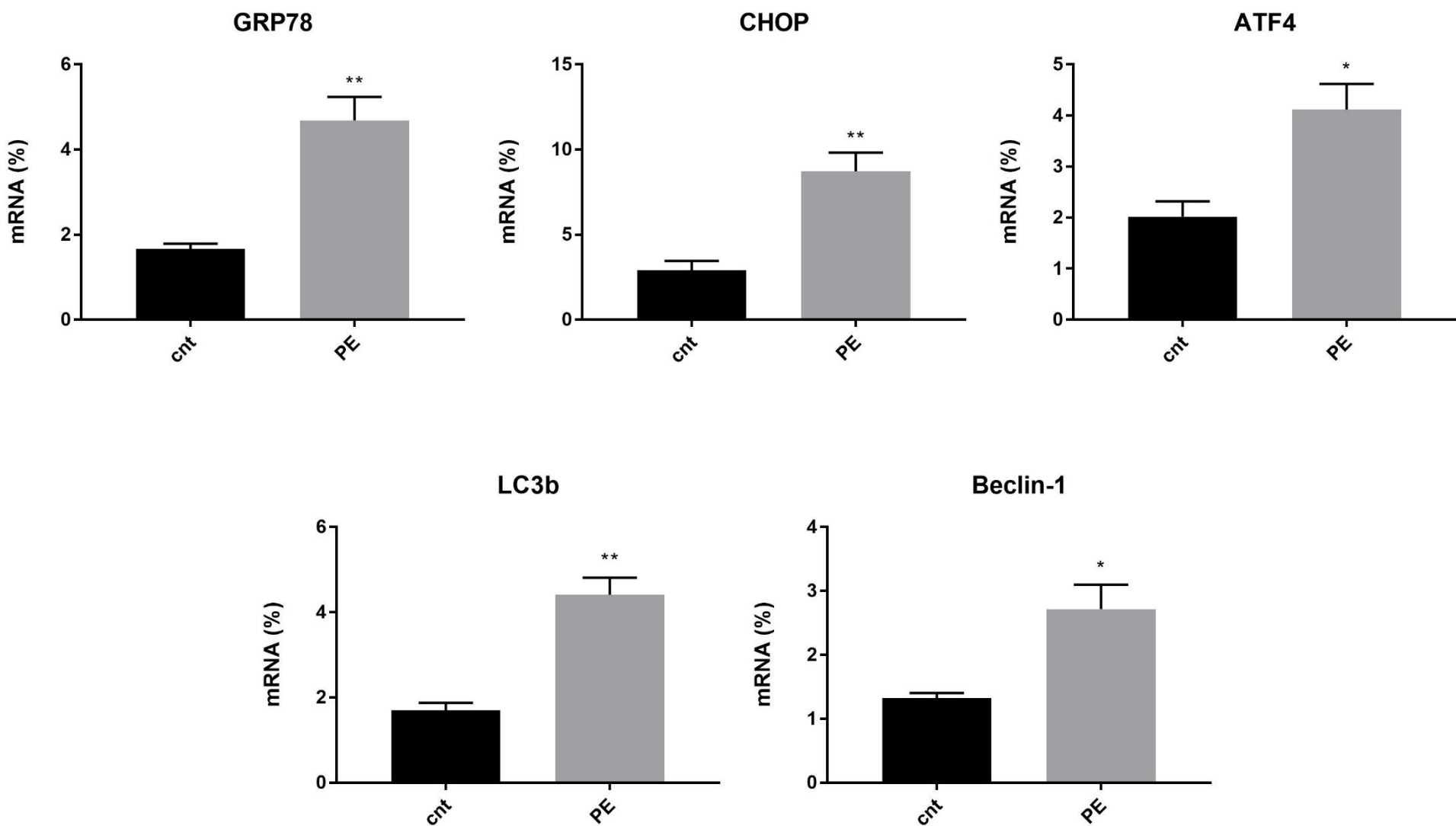

Figure S5

Supplement: Supplementary file 6 — Supplemental Figure 5 [file 41419_2019_1905_MOESM6_ESM.pdf]
